# Chemical Composition and Anxiolytic Potential of *Nectandra grandiflora* Essential Oil in Adult Zebrafish (*Danio rerio*): Behavioral, Molecular Docking, and ADMET Approaches

**DOI:** 10.1002/cbdv.71924

**Published:** 2026-11-14

**Authors:** Letícia Kelly Mesquita Rodrigues, Luiz Everson da Silva, Dayane Crystina da Silva Ceneviva, Wanderlei do Amaral, Jenifer Priscila de Araujo, Emmanuel Silva Marinho, Jessica Bezerra Maciel, Márcia Machado Marinho, Matheus Nunes da Rocha, Jane Eire Silva Alencar de Menezes, Hélcio Silva dos Santos

**Affiliations:** ^1^ Postgraduate Program in Biotechnology Sobral Ceará Brazil; ^2^ Postgraduate Program in Sustainable Territorial Development Federal University of Paraná Matinhos Paraná Brazil; ^3^ Department of Chemical Engineering Federal University of Parana Curitiba Paraná Brazil; ^4^ Postgraduate Program in Pharmaceutical Sciences Department Pharmacy Federal University of Paraná Curitiba Paraná Brazil; ^5^ State University of Ceara Graduate Program in Natural Sciences Fortaleza Ceará Brazil; ^6^ State University of Vale do Acaraú Chemistry Course Sobral Ceará Brazil

**Keywords:** anxiety, essential oil, terpene, zebrafish

## Abstract

*Nectandra grandiflora* essential oil was investigated for its chemical composition and neuropharmacological effects in adult zebrafish (*Danio rerio*), complemented by molecular docking and ADMET predictions. The oil was administered intraperitoneally at 4, 20, and 40 mg/kg (*n* = 6/group). No evident acute toxicity was observed after 96 h (LD_50_ >40 mg/kg). In the Light/Dark Test, only 4 mg/kg increased the time spent in the light compartment versus control (*p* < 0.0001), indicating an anxiolytic‐like effect. Flumazenil significantly reduced this response, supporting GABA receptor‐mediated neuromodulation. Conversely, none of the tested doses protected against PTZ‐induced seizures, differing significantly from diazepam (*p* < 0.0001). Molecular docking identified SEL as the compound with the most favorable predicted binding affinity to the GABA_A_ receptor, while ADMET analysis suggested potentially favorable pharmacokinetic properties; however, both findings require experimental validation to confirm their biological and pharmacological relevance. Overall, the oil showed anxiolytic‐like, but not anticonvulsant, activity under the evaluated conditions.

## Graphical abstract

Chemical profiling of *Nectandra grandiflora* essential oil revealed compounds with anxiolytic and anticonvulsant potential supported by docking and ADMET.

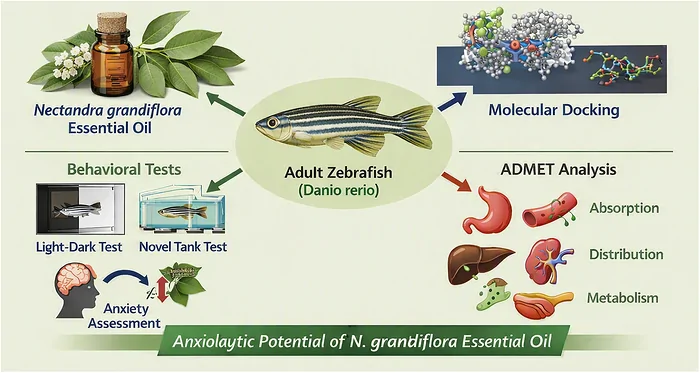

## 1 Introduction

Anxiety is considered a psychic disorder that affects thousands of people around the world. It is an emotion innate to the human being, but when in excess, it becomes a disease. It occurs through a high process of excitation of the central nervous system (CNS), generating unpleasant sensations of tension, fear, and restlessness, which are associated with the anticipation of something that may or may not happen [1]. It is estimated that in Brazil, in a survey carried out in 2017, at least 9.3% of the population had anxiety [2], with this number increasing even more during the SARS‐CoV‐2 pandemic (COVID‐19), about 44% of the Brazilian population felt more anxious about the whole situation the world was going through [3]. The best‐known anxiolytics are benzodiazepines and barbiturates. However, they have many associated toxic effects, such as sedation, drowsiness, dizziness, vertigo, mood swings, paranoia, irritability, and neurotoxicity [4, 5].

To search for new drugs with anxiolytic effects, several experimental animal models are available for the initial preclinical phase in vivo. Each has its particularities, advantages, and disadvantages. For example, Zebrafish (*Danio rerio*) has been widely used recently, as it has a high reproduction rate, ease of handling, small size, transparency, external fertilization, and low cost [6, 7]. Furthermore, its genetic homology with that of humans is 76%, with 82% for disease‐related genes. Compared to other animal models, such as mice, which are widely used, their genetic homology is 84% about living beings, a value very close to that of Zebrafish [8].

The Lauraceae family stands out for its composition of metabolites that have demonstrated biological activity, including alkaloids, neolignans, sesquiterpenes, phenylpropanoids, flavonoids, and tannins. It is also noteworthy for being used economically in the form of essential oils in cooking, papermaking, carpentry and construction, in the chemical industry and in folk medicine [9]. Lauraceae species are of great importance in the timber and cosmetics industries and there are reports of their use in folk medicine, with tonic, antirheumatic, antifungal, antimicrobial and diuretic actions, among others. Among the genera of this botanical family with the largest number of species and great economic importance are: *Endlicheria*, *Ocotea*, and *Nectandra*.

*Nectandra grandiflora* Nees is a native species endemic to Brazil. It is popularly known as canela‐amarela or caneleira and canela or canela‐fedida. The infusion of its leaves is used in folk medicine to treat digestive problems. The ethanolic extract of *N. grandiflora* leaves contains glycosylated flavonoids (kaempferol 3‐O‐β‐ramnoside and quercetin 3‐O‐β‐ramnoside), which exhibited antioxidant activity, inhibiting the oxidation of β‐carotene. In another study, they isolated protocatechuic acid and the flavonoids afzelin and quercetrin, which showed free radical scavenging activity against DPPH. The essential oil (EO) of the leaves has already been described as containing eugenol, a constituent with recognized antimicrobial and anaesthetic activity. In addition, antiparasitic activity and low larvicidal activity have been described for the EO [10].

The objective of this study was to analyze the chemical composition of the essential oil of the *N. grandiflora* and to assess, through in vivo experiments using Zebrafish (*Danio rerio*) as a study model, the anxiolytic biological activity of the essential oil of the *N. grandiflora*.

## 2 Results and Discussion

### 2.1 Chemical Composition of the Essential Oil of *Nectandra grandiflora*

Studies into native species with therapeutic potential are becoming increasingly important. Knowing the chemical composition and possible biological activities related to essential oils from native natural resources increases the possibility of creating protocols for the sustainable use of biodiversity. Table 1 shows the results of the composition of the study species collected in Paraná in the winter of 2018 [11].

**TABLE 1:** Chemical composition of essential oil (≥ 0.1%) from samples of *N. grandiflora* (OFNg) leaves collected in winter at RPPN Butuguara, Palmeira, PR, 2018.

Al	Theoretical Al	IK	Compound	%
1495	1471	1473	4,5‐di‐epi‐Aristoloquene	1,28
1514	1496	1496	Valencene	3,23
1522	1500	1500	Biciclogermancrene	3,65
1561	1542	1543	δ‐Cuprenene	1,30
1559	1552	1660	Germacrene B	0,85
1658	1684	1660	Dihidrofukinone epóxide	5,25
1689	1665	1666	Selin‐11‐en‐4‐α‐ol	5,64
1779	1732	1733	Zerumbone	8,69
1784	1733	1734	Isobiciclo germacrenal	4,07
1806	1837	1806	(+)‐Dihidrofukinone	49,10
1848	1808	1808	Eremofilan‐11‐en‐10‐ol	13,22
1917	1896	1896	Rimuene	2,08
2024	2042	2043	Kaurene	1,64
Total of identified compounds (%)				100%

Al = calculated retention index; Theoretical Al = literature retention index; IK = Kovats index.

*Source*: Prepared by the author.

Previous studies have shown a predominance of sesquiterpenes and oxygenated sesquiterpenes. A study conducted with essential oil collected in Rio Grande do Sul showed a predominance of the sesquiterpenes dehydrofukinone (DHF), dehydrofukinone epoxide (DFX), eremofil‐11‐in‐1‐ol (ERM) and selin‐11‐in‐4‐α‐ol (SEL). The anxiolytic effect was investigated for both the essential oil and the isolated compounds [12].

### 2.2 Acute Toxicity 96 h

Receiving increasing attention in the academic world, zebrafish, an animal model proposed to be used as the first contact of a substance with an organism, is present in various studies, including toxicity experiments that can be carried out at embryonic, larval, and adult stages [13, 14]. The advantages that make zebrafish so popular are its development, reproduction, handling, cost‐effectiveness, and, notably, its genomic similarity to humans, as well as its almost immediate response to external and internal stimuli, providing concise and rapid results [15].

Adult zebrafish are used to conduct acute toxicity tests on substances under study. The essential oil of *N. grandiflora* (OEPL37) did not induce evident acute toxicity in adult zebrafish during the 96‐h observation period; there was insufficient mortality to determine an LD_50_ within the tested dose range (LD_50_> 40 mg/kg), nor were macroscopic anatomical changes observed. No mortality or evident behavioral abnormalities attributable to the vehicle were observed.

The study by Barbas [16] et al. evaluated the 48‐h mortality rate of the essential oil of *N. grandiflora* at various concentrations in juvenile tambaqui fish (*Colossoma macropomum*), and the result showed no mortalities.

### 2.3 Assessment of Locomotor Activity (Open Field Test—OFT)

Zebrafish are evaluated through their behavior, swimming speed, and the quantification of their movement. Thus, locomotor activity is of great importance for analyzing and measuring the intensity of the action of a substance that will act on the central nervous system of adult zebrafish, providing the necessary stimulus to alter the animal's naturally anxious behavior, where it is expected to display actions consistent with the anticipated results [17, 18].

The open field test is appropriate as it designates certain parameters that will take into account the animal's swimming activity, as well as its movement, to assess whether locomotion is restricted, resulting in a more static animal, or if its swimming continues to show a regular pattern [19]. We adapted the open field test in Petri dishes, proposed by Ahmad and Richardson [20], to evaluate the locomotor activity of adult zebrafish under the action of analgesic drugs [21].

The lowest dose showed similarity to the control DZP and differed from the control DMSO 3% (****p < 0.0001), causing an effect on the animals' behavior, that is, leading to a significant reduction in swimming activity, indicating locomotor suppression (Figure 1). The 20 and 40 m/kg doses were statistically different from the DZP group (##*p* < 0.01).

**FIGURE 1:**
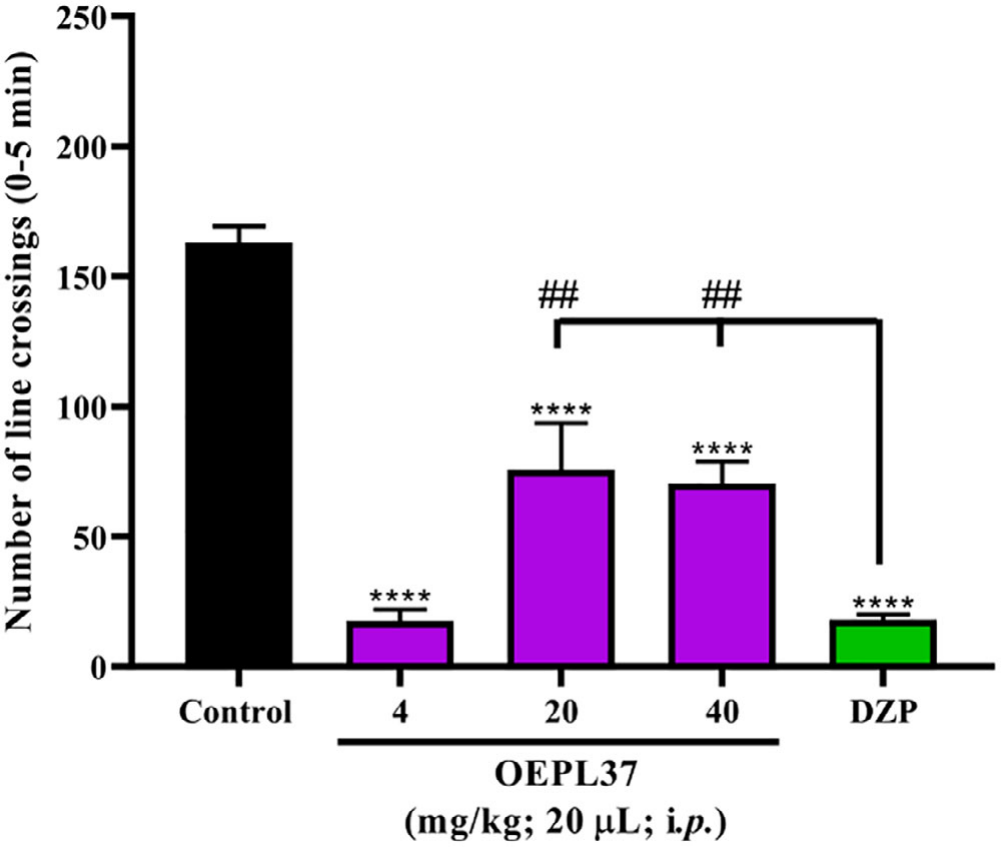
Effect of *N. grandiflora* oil on the locomotor behavior of adult zebrafish in the Open Field Test (0–5 min). The values represent the mean ± standard error of the mean for six animals/group; One‐Way ANOVA followed by Tukey's test: (****p < 0.0001 vs. Control; ^##^ *p* < 0.01 vs. DZP).

The anesthetic and sedative effects of *N. grandiflora* are well established for tambaqui (*Colossoma macropomum*) and jundiá (*Rhamdia quelen*), two economically relevant fish species in Brazil [16, 22, 23]. It has been shown that the main component of the essential oil of *N. grandiflora*, DHF, has sedative activity in fish [22].

### 2.4 Anxiolytic Assessment (Light/dark Test—LDT)

Zebrafish, similar to humans and several other species, naturally exhibit anxious behavior, which results in their preference for areas with reduced light. This factor is the main parameter that drives the Light–Dark test. Based on this behavioral pattern, the state of relaxation or calmness induced by the substance under investigation is assessed by the amount of time the animal spends in the brighter area of the tank. Other factors are also considered, such as the time required for the fish to move to the dark side of the container and the number of alternations between the light and dark sides of the tank [19].

Looking at the graph shown in Figure 2, the 20 and 40 mg/kg doses of the OEPL37 sample revealed different results compared to the behavior exhibited by the positive control Diazepam (#*p* < 0.05, ###*p* < 0.001), indicating the absence of a potential anxiolytic effect, with animals spending less time in the light zone of the tank. Only the 4 mg/kg dose showed similarity to DZP and a difference from the negative vehicle control DMSO 3% (####*p* < 0.0001), suggesting a possible anxiolytic effect. To further investigate the anxiolytic effect, the most effective dose, 4 mg/kg, was used. The anxiolytic response observed only at 4 mg/kg suggests a non‐linear dose–response relationship. This effect does not appear to result from greater sedation at the higher doses, since the open‐field test showed a more pronounced reduction in locomotor activity at 4 mg/kg. Therefore, increasing the dose of the essential oil did not enhance its anxiolytic activity. Considering the multicomponent nature of essential oils, interactions among their constituents at different concentrations may contribute to this behavioral response.

**FIGURE 2:**
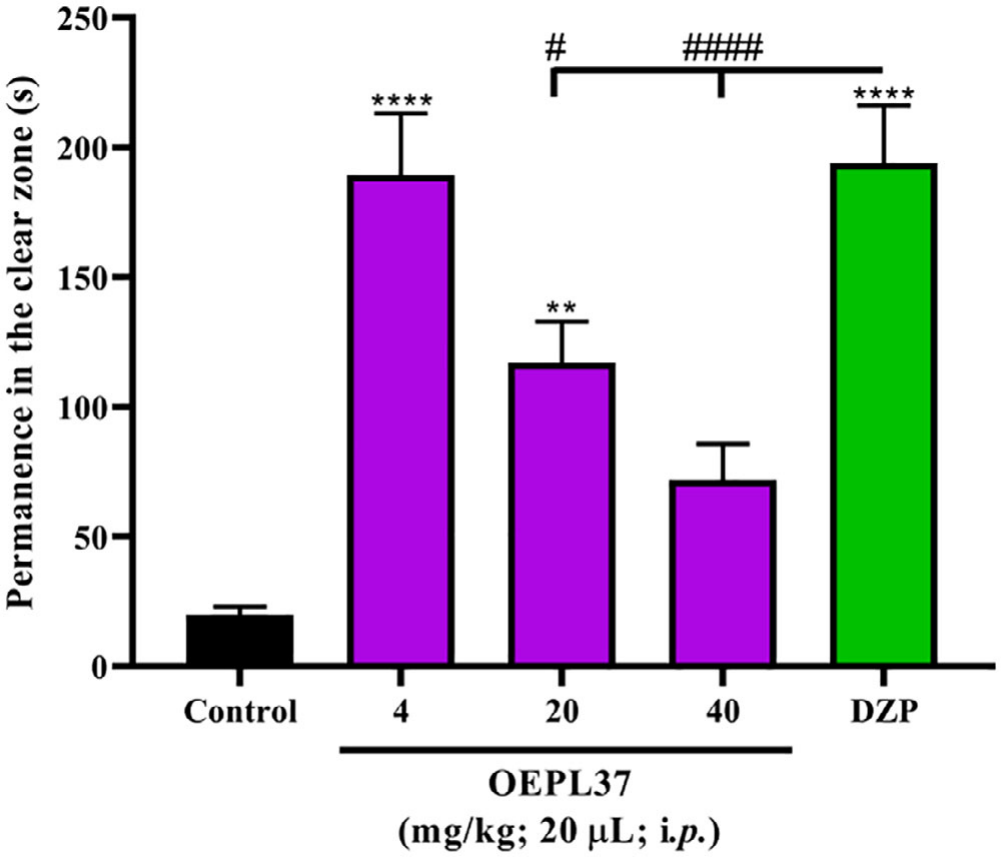
Effect of *N. grandiflora* oil on zebra fish anxiety in the Light/Dark test (0–5 min). The values represent the mean ± standard error of the mean for six animals/group; One‐Way ANOVA followed by Tukey's test: (**p < 0.01, ****p < 0.0001 vs. Control; ^#^ *p* < 0.05, ^####^ *p* < 0.0001 vs. DZP).

In the study by Garlet [12] et al. the essential oil of *N. grandiflora* and the four isolated compounds (DHF, DFX, ERM, SEL) demonstrated anxiolytic effects in mice. This was evidenced by an increased time spent in the center of the open field and elevated arms tests, typical of less anxious animals, as well as a reduction in the time spent in the more peripheral zones (usually associated with anxiety). Furthermore, two of the isolated compounds, ERM and SEL, were particularly effective at low doses (such as 6 mg/kg for ERM and 4 mg/kg for SEL), suggesting that these compounds, even in lower concentrations in the essential oil, significantly contributed to the observed anxiolytic effects.

### 2.5 Assessment of GABAergic Neuromodulation (GABAA receptor)

Gamma‐aminobutyric acid (GABA) is a chemical messenger that transmits information from one neuron to another, regulating the nervous system. It is considered the main inhibitory neurotransmitter in the central nervous system (CNS), being released by GABAergic neurons and binding to specific receptors on other neurons. Among its receptors, the gamma‐aminobutyric acid type A (GABAA) receptor is the most widely distributed fast‐acting receptor in the CNS of mammals. GABAA receptors are pentameric ion channels that span the membrane and exhibit high heterogeneity among their subunits. When these receptors are activated by the neurotransmitter GABA, they allow chloride ions to enter the neurons, resulting in the hyperpolarization of these cells, making them less responsive to excitatory neurotransmitters. In other words, these receptor neurons undergo a decrease in neuronal conduction, leading to the inhibition of the nervous system. GABAA receptors are targeted by several pharmacological groups with anesthetic and sedative properties [24, 25].

Thus, to investigate the mechanism of action of OEPL37 (4 mg/kg), which showed an anxiolytic effect (best effective concentration in the Light/Dark test), we analyzed the neuromodulation mechanism of anxiety via GABAA. In general, it is expected that during the neuromodulation test, the antagonist flumazenil will block the GABAergic receptors, preventing the sample from acting on them. Therefore, the animals, which naturally exhibit anxious behavior, would continue to display such behavior.

In this context, the OEPL37 sample (4 mg/kg, Figure 3) showed its anxiolytic effect neuromodulated by the GABAA receptor as there was a statistically significant difference between the sample and the sample with the antagonist flumazenil (OEPL37 vs Fmz + OEPL37; Figure 3). This means that the anxiolytic effect was blocked by flumazenil, indicating the involvement of the sample in this channel.

**FIGURE 3:**
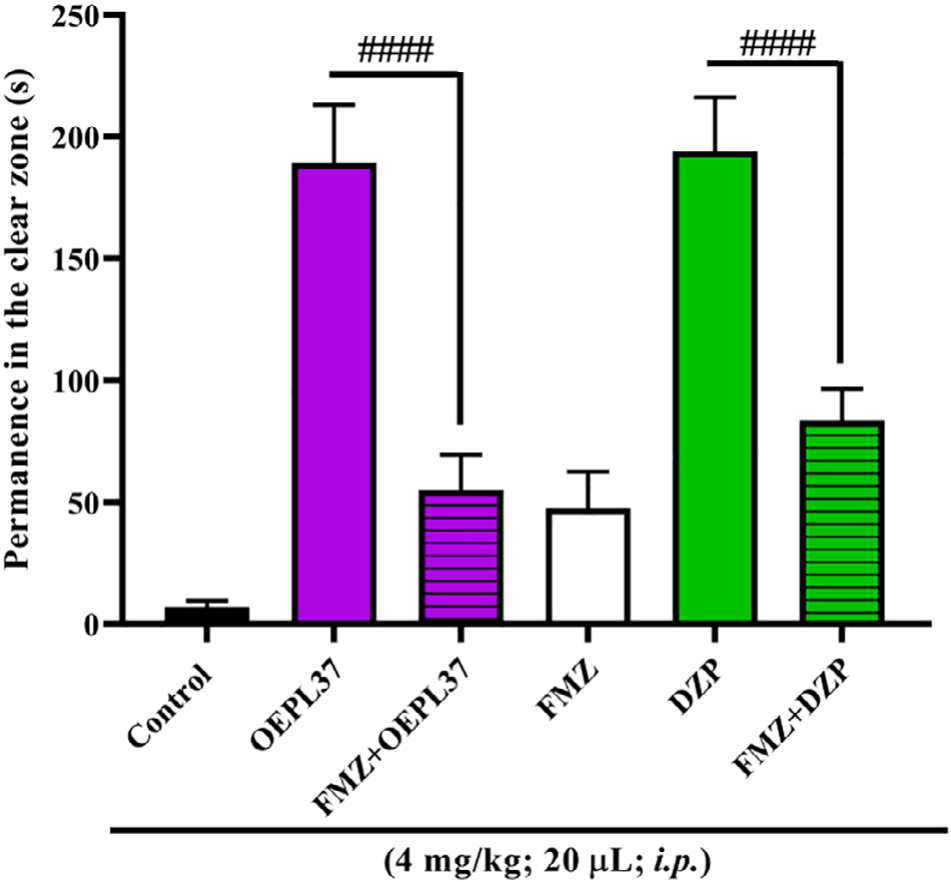
Effect of *N. grandiflora* oil on GABAergic neuromodulation in zebrafish in the Light/Dark test (0–5 min). The values represent the mean ± standard error of the mean for six animals/group; Two‐way ANOVA followed by Tukey's test. (####*p* < 0.0001 OEPL37 vs. OEPL37+DZP, ####*p* < 0.0001 DZP vs. FMZ+DZP).

Importantly, although OEPL37 reduced locomotor activity in the Open Field Test, the significant increase in the time spent in the light compartment at 4 mg/kg indicates that the behavioral response in the LDT was not solely dependent on locomotor suppression. This interpretation is further supported by the significant reduction of this effect following flumazenil pretreatment, indicating the involvement of GABAA receptor‐mediated neuromodulation.

Although reduced locomotor activity was observed, the increased time spent in the light compartment and its sensitivity to flumazenil support an anxiolytic‐like response involving GABA receptor‐mediated neuromodulation.

The study by Garlet [12] et al. suggests that the anxiolytic effect of the essential oil of *N. grandiflora* and its compounds is mediated, at least in part, by GABAA receptors, particularly by the benzodiazepine site of these receptors. When the antagonist FMZ was administered, the anxiolytic effect was reduced, suggesting that the modulation of these receptors is crucial for the observed effects. Additionally, some compounds (DFX, ERM, and SEL) also seem to act independently of the interaction with the benzodiazepine site by modulating neuronal calcium influx, which may also contribute to the reduction of anxiety.

Phootha et al. (2022) [26] also confirm that sesquiterpenoids extracted from *N. grandiflora* Ness are involved in the control of anxiety through GABAA receptors. Similarly, Batista et al. (2024) [24] demonstrated anxiolytic‐like effects of an essential oil in adult zebrafish associated with GABA‐mediated neuromodulation, reinforcing the relevance of this pathway in the neuropharmacological effects of essential oils.

### 2.6 Anticonvulsant Evaluation

PTZ acts on the GABAA receptor, blocking the Cl channel, and is commonly used to induce the progressive development of seizures in animal models. Repeated treatment with PTZ is associated with biochemical and histological changes similar to those observed in epileptic people, which are the main responsible for the cognitive and emotional behavioral impairment demonstrated in animal models [24].

The anticonvulsant effect of OEPL37 and Diazepam on PTZ‐induced seizures in adult zebrafish was investigated. The results demonstrate a significant difference between all the doses tested and the positive control DZP (####*p* < 0.0001) (Figure 4) in all stages (Figure 4A‐‐C), showing the non‐occurrence of a potential anticonvulsant effect, that is, it was observed that the animals presented a shorter latency time for the onset of seizures in all stages.

**FIGURE 4:**
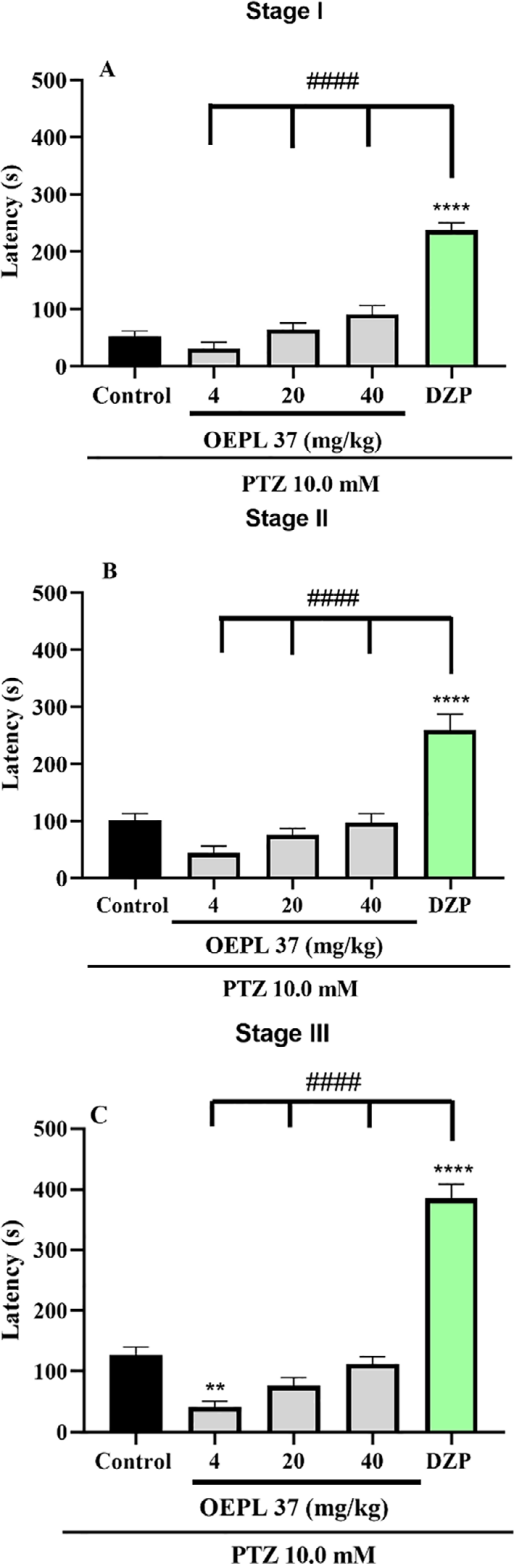
Anticonvulsant test of OEPL37 (A) Stage I, (B) Stage II, (C) Stage III. Anticonvulsant effect of the sample in adult zebrafish (Danio rerio) from PTZ‐induced seizure. Vehicle control (DMSO 3%; 20 µL; i.p.); Dzp – Diazepam (4 mg/kg; i.p.). Values represent mean ± standard error of the mean (S.E.M.) for six animals/group; ANOVA followed by Tukey. (**p < 0.01, ****p < 0.0001 vs. Control; ####*p* < 0.0001 vs. DZP).

Although the essential oil showed GABAA, related anxiolytic activity, this neuromodulatory effect was not sufficient to protect against PTZ‐induced seizures under the experimental conditions evaluated. Anxiolytic and anticonvulsant responses do not necessarily require the same degree of GABAergic modulation, and the robust neuronal hyperexcitability induced by PTZ may require a stronger or distinct pharmacological effect. Thus, the present findings indicate a behavioral anxiolytic response without a corresponding anticonvulsant effect at the doses evaluated.

### 2.7 Molecular Docking Analysis

After completing the independent molecular docking simulations, the compounds DFX, DHF, ERM, and SEL exhibited predicted binding affinity energies of ≤ −6.0 kcal/mol for the GABAA receptor, indicating potentially favorable ligand–receptor interactions. Among these compounds, SEL showed an affinity energy of −8.12 kcal/mol, comparable to that predicted for diazepam (DZP; −8.44 kcal/mol) (Figure 5a). Redocking validation demonstrated an RMSD of approximately 0.35 Å between the redocked DZP structure (green) and the cocrystallized ligand (grey) in the GABAA receptor structure (PDB ID: 6HUP) (Figure 5b), supporting the reproducibility of the docking protocol at the benzodiazepine‐binding site [26]. All simulations met the predefined RMSD threshold of < 2.0 Å, indicating consistency in the selection of the predicted binding poses (Table 2).

**FIGURE 5:**
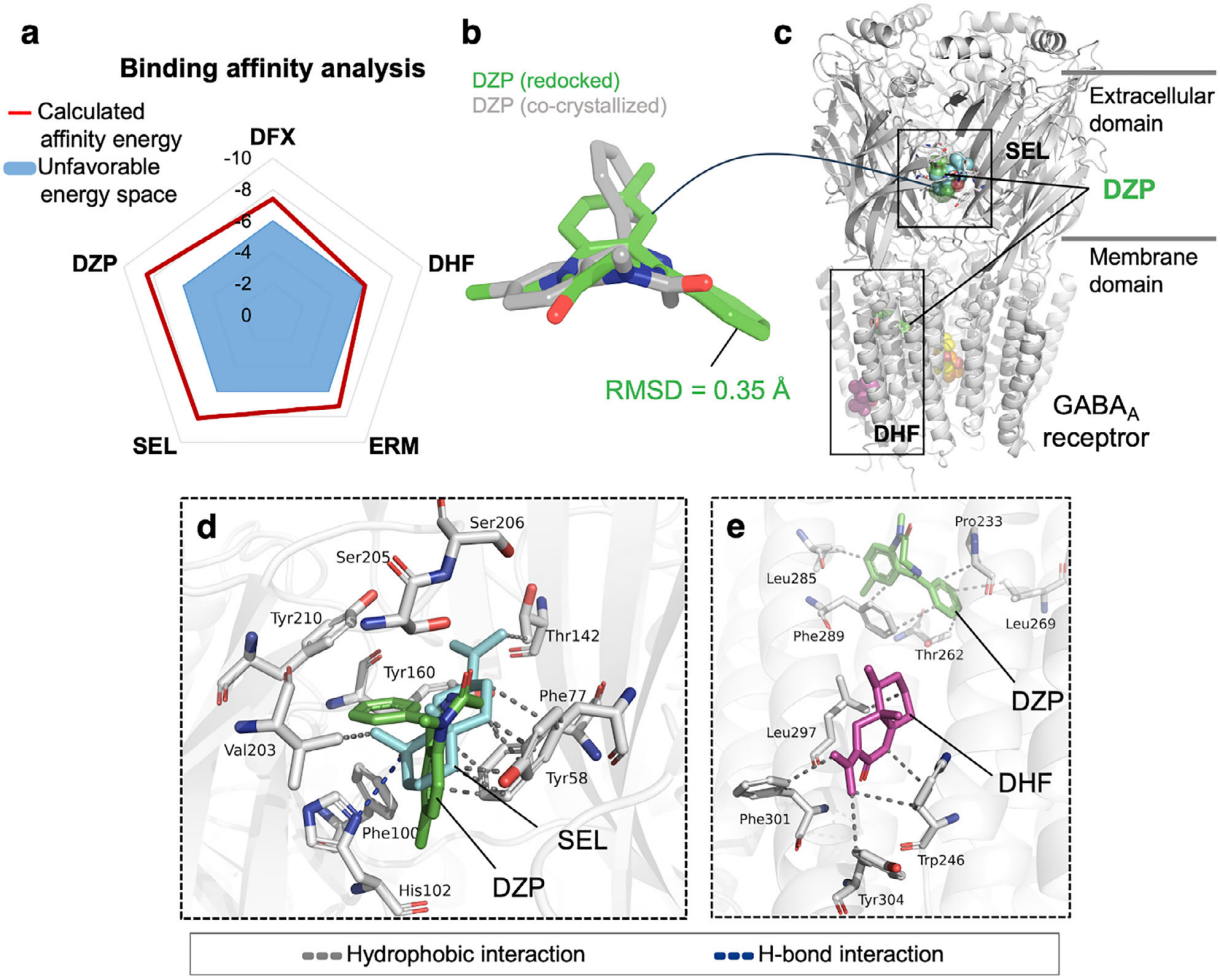
(A) Radar plot showing the distance of the calculated affinity energy values for the compounds DFX, DHF, ERM, and SEL upon binding to the GABAA receptor in unfavorable space (> –6.0 kcal/mol). (B) Redocking of the agonist DZP (green) relative to the co‐crystallized DZP (gray) in PDB code 6HUP. (C) Three‐dimensional visualization of the binding of the ligands DFX, DHF, ERM, and SEL to the binding domains of the agonist DZP at the GABAA receptor. Analysis of ligand‐receptor interactions of the ligands (D) SEL and (E) DHF to the binding domains of DZP at the GABAA receptor.

**TABLE 2:** Results of molecular docking simulations expressed in affinity energy, RMSD, and ligand‐receptor interactions, for the ligands DFX, DHF, ERM, and SEL targeting the GABAA receptor.

Compound	Energy	RMSD	Type	Residue	Distance
DFX	−7.42	1.79	Hydrophobic	Ala252	3.44
				Ala252	3.79
				Ile255	3.46
				Thr256	3.71
				Val257	3.82
				Val257	3.71
				Leu259	3.79
			H‐bond	Thr256	2.42
DHF	−6.17	1.49	Hydrophobic	Trp246	3.79
				Trp246	3.69
				Leu297	3.35
				Phe301	3.70
				Tyr304	3.67
ERM	−7.15	1.67	Hydrophobic	Ala252	3.89
				Thr256	3.87
				Val257	3.94
				Val257	3.72
				Val257	3.47
				Leu259	3.82
				Leu259	3.68
				Leu264	3.86
				Leu274	3.65
SEL	−8.12	1.85	Hydrophobic	Tyr58	3.50
				Tyr58	3.22
				Phe77	3.89
				Phe77	3.78
				Phe77	3.67
				Phe77	3.60
				Phe77	3.60
				Phe77	3.91
				Thr142	3.63
				Val203	3.58
DZP	−8.44	0.35	Hydrophobic	Tyr58	3.52
				Phe77	3.47
				Phe100	3.83
				Phe100	3.92
				Tyr160	3.56
				Val203	3.95
				Tyr210	3.38
				Tyr210	3.86
			H‐bond	Ser205	2.94
				Ser206	3.16
			Halogen bond	His102	3.82

The docking results further suggested that SEL may occupy a binding pocket in the extracellular domain of the GABAA receptor that overlaps with the DZP‐binding site, whereas DHF was predicted to bind to a distinct site in the transmembrane region (Figure 5c). Analysis of the predicted ligand–receptor interactions indicated that SEL could establish hydrophobic contacts with residues also involved in DZP binding, including Tyr58 and Phe77, through its bicyclic structure, as well as with Thr142 and Val203 through its terminal methyl groups (Figure 5d). In contrast, DHF was predicted to interact with the transmembrane domain without sharing the same residue contacts identified for DZP (Figure 5e).

Although these findings suggest that SEL may have the structural potential to interact with the benzodiazepine‐binding site of the GABAA receptor and potentially interfere with ligand binding, the results must be interpreted cautiously. Molecular docking estimates binding affinity and predicts plausible ligand–receptor interactions; it does not directly demonstrate receptor activation, inhibition, or allosteric modulation. Therefore, the similarity between the predicted binding energies of SEL and DZP, as well as the overlap in their predicted binding sites, should not be interpreted as evidence of equivalent pharmacological activity or competitive binding. Further experimental investigations, including receptor‐binding and functional assays, are required to confirm these interactions and determine whether SEL modulates GABAA receptor activity [27].

### 2.8 PK/PD Study and Toxicity Prediction

#### 2.8.1 Medicinal Chemistry Evaluation

In a potential analysis of molecular lipophilicity, it is possible to observe that the compounds are essentially hydrophobic on their molecular surfaces (blue color spectra), favoring their affinity with organic solvents and biological tissues/membranes (Figure 6). The DFX compound has a polar surface area in the order of 29.60 Å^2^ associated with the epoxide (R—O—R) and carbonyl (C = O) groups (red color spectra), resulting in a logP in the order of 2.88 (Figure 6a) [28]. In contrast, the ERM (Figure 6c) and SEL (Figure 6d) compounds have a polar surface of 20.23 Å^2^, strongly influenced by the H‐bond donation (HD) potential of the —OH group, resulting in moderate logP indices with calculated values in the order of 3.2 and 3.27, respectively (Table 3). On the other hand, the DHF compound (Figure 6b) has the highest lipophilicity in this study (logP: 3.44), due to the lower polar surface of the C = O group in relation to groups for being an H‐bond acceptor (HBA^)^ [28].

**FIGURE 6:**
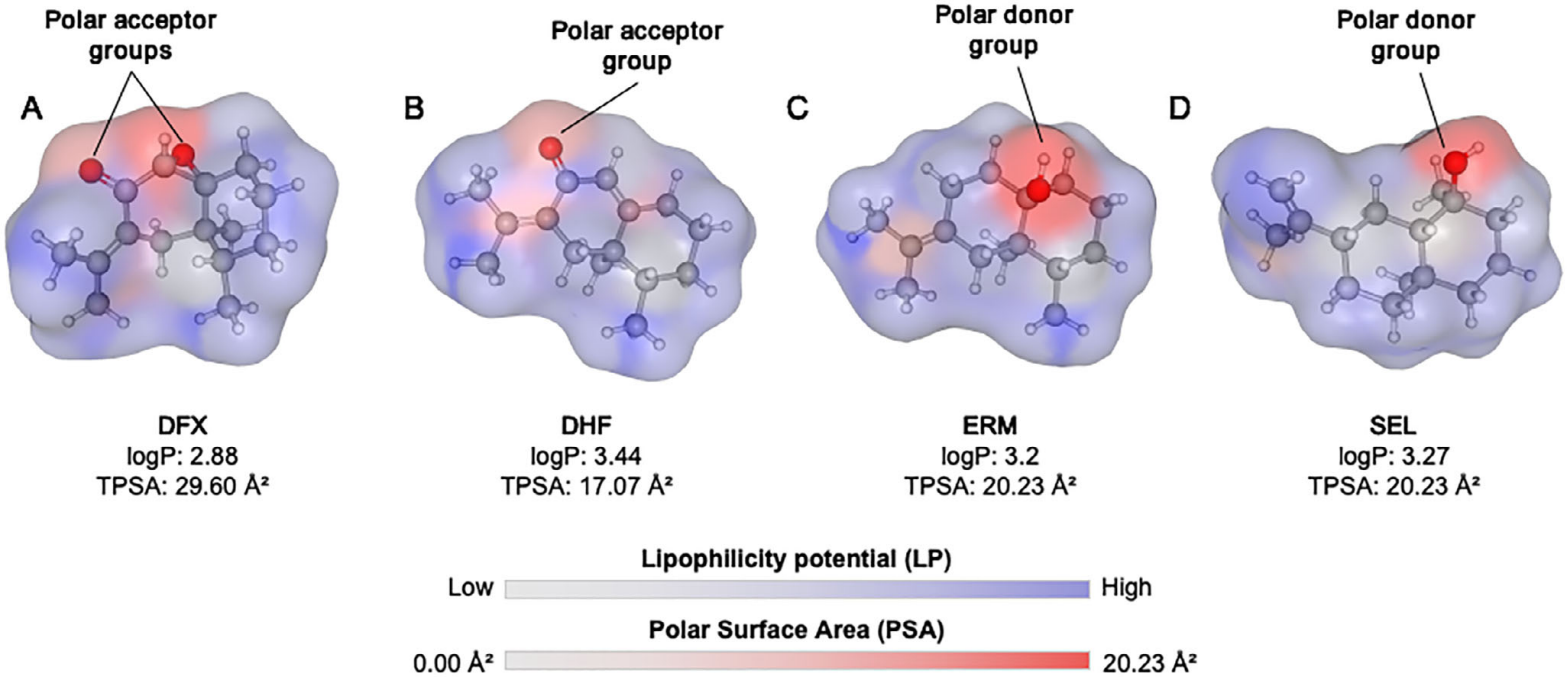
Surface map of molecular lipophilicity potential (MLP) for compounds (A) DFX, (B) DHF, (C) ERM, and (D) SEL.

**TABLE 3:** Predicted physicochemical and pharmacokinetic properties for the compounds DFX, DHF, ERM, and SEL.

Property	Compounds				Optimal
	DFX	DHF	ERM	SEL	
*Medicinal chemistry*					
logP	2.88	3.44	3.2	3.27	≤ 3.0
MW (g/mol)	234.16	218.17	222.2	222.2	200–600
nHA	2	1	1	1	0–5
nHD	0	0	1	1	2–10
TPSA (Å^2^)	29.6	17.07	20.23	20.23	20–130
nRot	0	0	0	1	0–10
nRing	3	2	2	2	0–3
Alert	2	1	1	1	0
QED score	0.48	0.56	0.61	0.67	≥ 0.6
*Pharmacokinetics*					
P_app,A→B_ Caco‐2 (in 10^−5^ cm/s)	2.69	2.63	2.18	1.90	≥ 10 × 10^−6^ cm/s
P_app,A→B_ MDCK (in 10^−5^ cm/s)	2.34	2.23	02.08	1.58	≥ 10 × 10^−6^ cm/s
P‐gp efflux	0.01	0.04	0.30	0.00	≤ 0.30
%F	0.90	0.83	0.86	0.86	> 50
HIA (%)	97.14	97.90	92.51	93.51	> 75
logBB	0.52	0.61	0.53	0.59	≥ 0.1
PPB (%)	88.78	90.98	93.99	95.31	≤ 90
CL_int,u_ (mL/min/kg)	12.45	10.41	15.06	14.85	≤ 15
HLM stability	0.88	0.98	0.86	0.87	> 0.75
*Toxicity*					
hERG blockers (%)	35.45	34.53	37.56	37.26	≤ 50
Ames (%)	44.23	43.07	44.12	44.02	≤ 50
DILI (%)	34.98	41.52	37.4	43.12	≤ 50
LD_50_ (‐log mol/kg)	1.88	2.13	2.15	1.94	≤ 1.0

Despite the alignment between low lipophilicity (logP < 3) and TPSA > 20.0 Å^2^ (Figure 7a), as estimated by Pfizer, Inc., for compounds with high pharmacokinetic viability, DFX has two structural alerts associated with the C ═ O groups conjugated to the α,β‐unsaturated system (Michael acceptor) and to the epoxide group, as interference or potentially reactive structures, reducing the QED to 0.48 (on a scale of 0.0 to 1.0). On the other hand, the ERM and SEL compounds have a total of two rings (nRing) and only one interference structure associated with the isolated alkene, fitting within the spectrum of drug‐like properties of QED, with scores > 0.60, raising the hypothesis of a favorable pharmacokinetic/pharmacodynamic profile (Figure 3b), while DHF may be much less polar than safer compounds in the Pfizer, Inc., classification system (Figure 7b).

**FIGURE 7:**
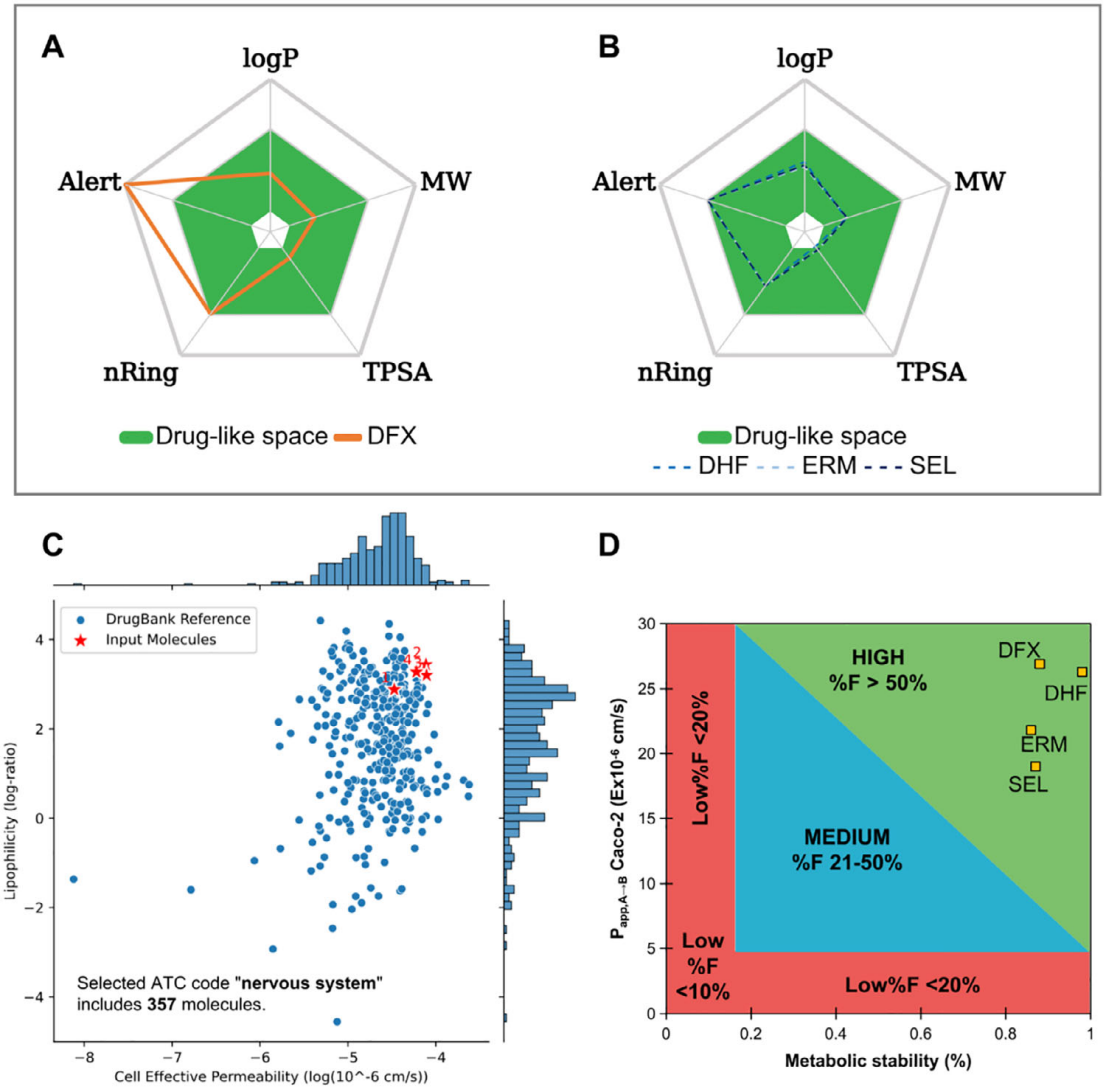
Oral bioavailability radar of compounds (A) DFX, (B) DHF, ERM and SEL, (C) estimation of lipophilicity and cell effective permeability guided by artificial intelligence in relation to compounds active in the nervous system from the DrugBank database and (D) alignment between permeability (Papp) Caco‐2 and metabolic stability to estimate oral bioavailability.

### 2.9 Pharmacokinetic Descriptors

The PK/PD prediction approach used here seeks to correlate physicochemical descriptors such as lipophilicity (logP) and polarity (TPSA) with the permeability, solubility, and systemic distribution of compounds. Compounds with low lipophilicity (logP ≤ 3) can be both highly permeable in more selective cell lines (P_app,A→B_ MDCK > 10 × 10^−6^ cm/s), such as the MDCK cell line, and metabolically stable in the human liver microsome (HLM) system.

The similarity test with drugs acting on the nervous system, which includes 357 active compounds deposited in the DrugBank database, revealed that the compounds DFX, DHF, ERM, and SEL reside in a physicochemical space formed by substances with high effective cell permeability, with logP_app_ > –5.0 cm/s, where the *P*_app_ values are in the order of 10^−6^ cm/s, and with moderate to high lipophilicity, where the logP values are > 2.8 (Figure 7c). This prediction corroborates the PAMPA profile predicted for the molecules, where metabolic stability and Caco‐2 cell permeability data were plotted, estimating the degree of oral bioavailability of the compounds (Figure 7c).

Prediction showed *P*_app,A→B_ values in Caco‐2 cells > 1.9×10^−^^5^ cm/s and are within a physicochemical stability range in human liver microsomes (HLM) > 0.8 (> 80%). These values are related to compounds with high cell permeability and low hepatic metabolism in the literature [29, 30]. The data suggest a pharmacokinetic profile consistent with slow release into the bloodstream and oral bioavailability (%F) > 50% (Figure 7d). Corroborating this, %F fractions > 0.8 were predicted, associated with more than 80% bioavailability of the non‐biotransformed molecular fraction by HLM in the bloodstream (Table 2). Furthermore, *P*_app,A→B_ values in MDCK cells > 1.5×10^−^^5^ cm/s suggest a possible effective permeability in more selective cell lines. In predicting absorption, the low probability of P‐gp‐dependent efflux (< 30%) and the predicted rate of HIA > 92% suggest gradual absorption in the gastrointestinal tract (Table 2) [31].

The low lipophilicity profile (logP < 3) of DFX is consistent with better distribution in blood plasma [28]. The ADMET prediction showed a plasma protein binding (PPB) value of approximately 88.78% (Table 2) for this compound. However, logP > 3 may be associated with a wider distribution of SEL in the CNS, since it showed a predicted logBB of 0.59 (Table 2).

### 2.10 Site of Metabolism and Reactivity

The prediction of the site of metabolism showed that the compounds DFX and DHF have low probability of being biotransformed in first‐pass metabolism. On the other hand, it was possible to identify signs of the presence of possible metabolism sites in the ERM and SEL derivatives (Figure 8). In this prediction, it was noted that the terminal methyl groups (—CH3) C.15 and C.16 of the ERM compound are more sensitive to phase I metabolism, with a probability of around 0.80 (on a scale of 0.0 and 1.0), forming metabolites based on aliphatic hydroxylation and olefin oxidation, which are more polar and more susceptible to excretion in plasma (Figure 8a), with a predicted CL_int,u_ of 15.06 mL/min/kg (Table 2). SEL, on the other hand, has a terminal alkene that can undergo epoxidation by the action of CYP450s, with a probability of 0.54 at atom C.2, as well as an aliphatic hydroxylation site in the terminal —CH3 group (C.1), with a probability of 0.45 (Figure 8b), which may be related to the rapid plasma clearance predicted by the CL_int,u_ value of 14.85 mL/min/kg.

**FIGURE 8:**
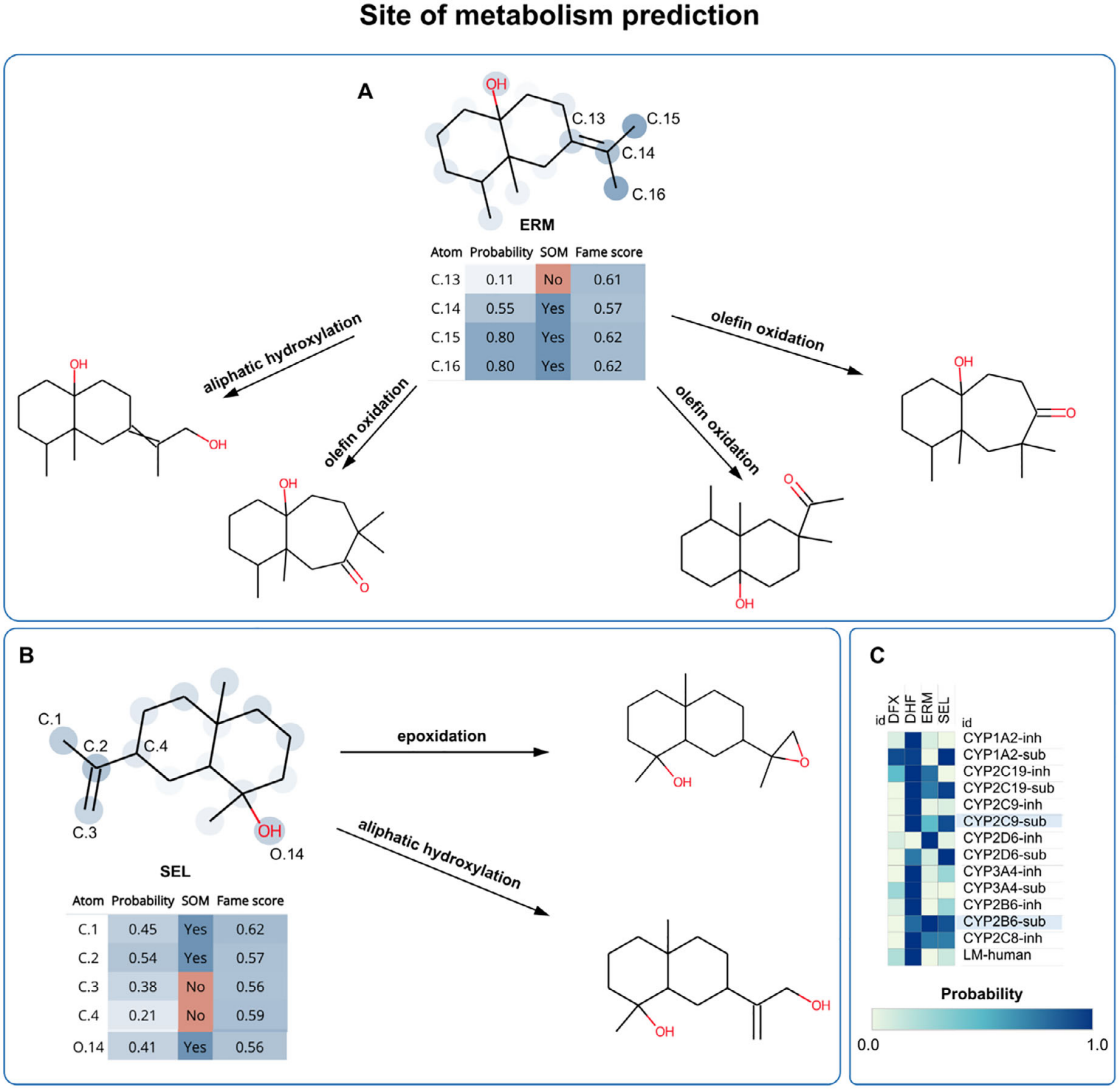
Prediction of the site of metabolism of the metabolically less stable compounds from this study: (A) ERM and (B) SEL. (C) susceptibility heatmap of interaction with CYP450 isoforms in the human liver microsome system.

The predictive PK/PD test guided by an ensemble model that combines multiple machine learning models suggests that the ERM and SEL compounds may be substrates of the CYP2C9 and CYP2B6 isoforms (Figure 8c), without inducing inhibition of the same, indicating that the compounds can be biotransformed by these pathways, affecting their distribution states, with PPB > 93% (Table 2). On the other hand, the DFX and DHF compounds may be metabolically more stable, with CL_int,u_ < 13.0 mL/min/kg and PPB < 92% (Table 2). DHF may be an inhibitor of various forms of CYP450s, which may affect the oral bioavailability of co‐administered substances, while DFX has low affinity with CYP450 isoforms in its epoxidized form (Figure 8c).

The 2D susceptibility map showed that the compounds DFX, DHF, ERM, and SEL exhibit signs of negative contribution (red color spectra) to acute oral toxicity prediction (Figure 9). DFX showed two structural alert signals associated with a Michael acceptor, that is, the carbonyl group conjugated to an alkene (─C═C─C═O), as well as a three‐atom heterocyclic group (Figure 9a), indicated as a negative structural contribution (red color spectra). In this test, the compound presents a relative probability of being non‐toxic with a confidence level of 64.0% since the carbon atoms farthest from the most electronegative groups constitute positive contributions (green color spectra) (Figure 9a). The most reliable test is associated with DHF, with a non‐toxicity decision with a confidence level of around 72.0% (Figure 9b). The susceptibility map showed that, despite the negative contribution associated with a Michael receptor of the type ─C═C─C═O (red color spectra), the positive contributions are aligned with the binary decision (green color spectra) (Figure 9b).

**FIGURE 9:**
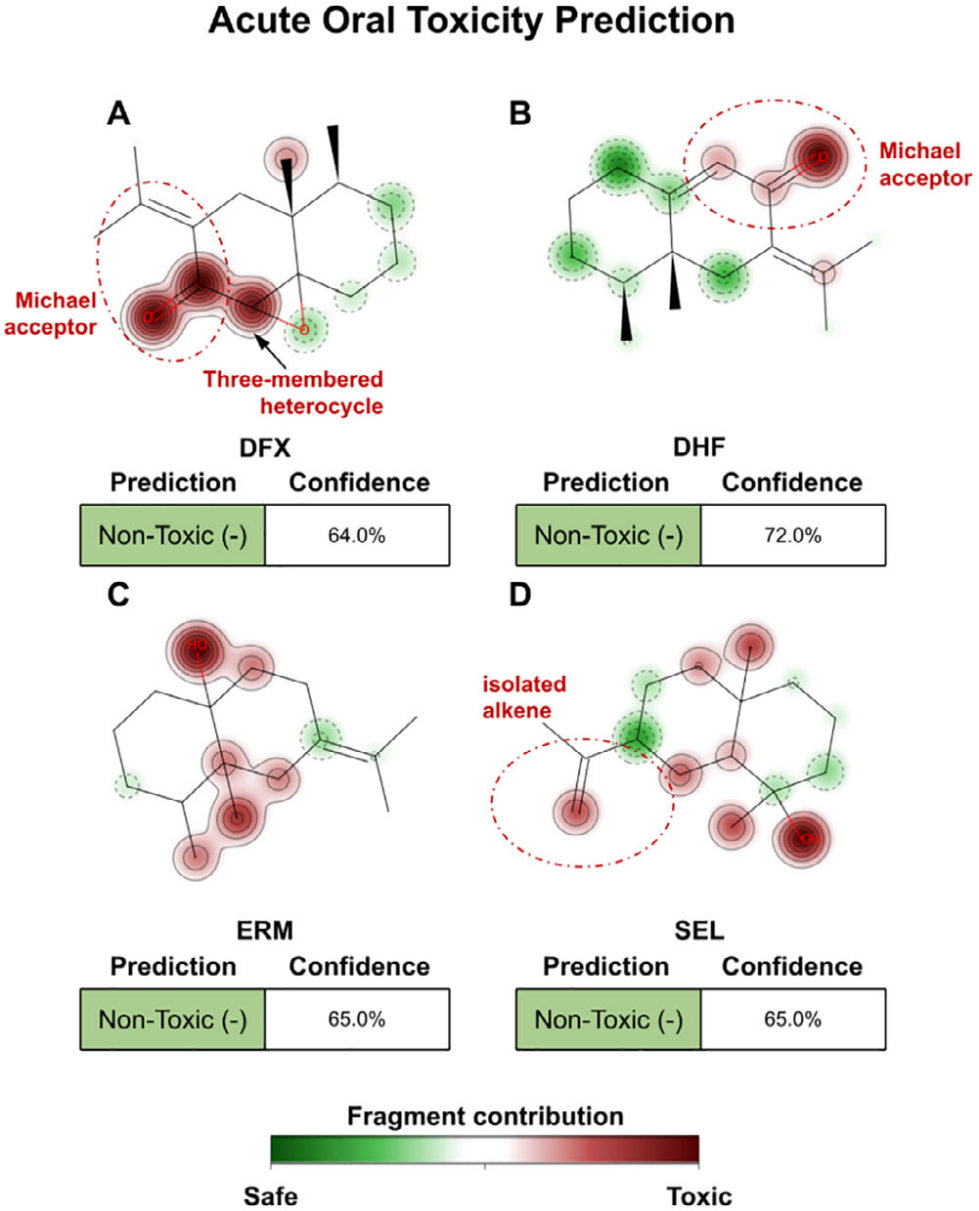
Predicted acute oral toxicity susceptibility map for compounds (A) DFX, (B) DHF, (C) ERM, and (D) SEL.

The compounds ERM (Figure 5c) and SEL (Figure 9d) presented a structural alert associated with the isolated alkene, but as a negative contribution in the SEL compound (Figure 9d), both with confidence in the order of 65.0%. Isolated alkenes are targets for epoxidation, which can increase the reactivity of molecules towards proteins and DNA, generating genotoxic responses [32].

Given the probabilities and the degree of confidence < 75%, Ames mutagenicity risks are predicted to be around 44% probability, and around 40% for the drug‐induced liver injury (DILI) descriptor (Table 2). However, LD_50_ values < 2.0 in –log(mol/kg) were predicted, estimating greater safety for the DFX and SEL compounds, indicating a low risk of acute oral toxicity (Table 2).

## 3 Experimental Section

### 3.1 Drugs and Reagents

The following substances were used: Diazepam (DZP, Neo Química), Flumazenil (Fmz; Sandoz), Dimethylsulfoxide (3% DMSO; Dynamic).

## 4 Materials and Methods

Before injection, the sample was diluted to 1% with dichloromethane (99.9%). The gas chromatography analysis was carried out on a Shimadzu GCMS‐TQ8040, split 90 chromatograph coupled to a mass spectrometer. GC‐MS measurements were carried out using an Rtx‐5MS non‐polar capillary column (5% diphenyl + 95% dimethyl polysiloxane, 30 m × 0.25 mm i.d. × 0.25 µm film thickness), operated under programmed temperature conditions from 60°C to 250°C at 3°C per minute. The carrier gas was helium with a flow rate of 1.02 mL min^−1^ and a linear velocity of 36.8 cm s^−1^ and the column head pressure was constant at 59 kPa. The injection port was set at 250°C, with an injection volume of 1.0 µL in split mode (ratio 1:10) [25]. After the chromatography procedure, the chromatograms were analyzed in a program called GCMS Postrum Analysis, from which the components present in the essential oil samples were identified. The data found was compared with the results of the specific bibliography [33].

### 4.1 Botanical Material

The fresh leaves of the species were collected for the extraction of essential oil samples in the municipality of Palmeira (25°38'S; 49°31'W), state of Paraná, Brazil, in the fall of 2018. To collect and transport the plant material in the reserve, a license was issued by the Environmental Institute of Paraná (number 284). Institutional registration was also carried out with the Genetic Heritage Management Council—CGEN of the National Ministry of the Environment (No. A0617A4). Specimens of the *N. grandiflora* plant were located in the field, marked with a GPS, exsiccated for taxonomic identification, photographed and 1 kg of dry material collected for the extraction of essential oil. The botanical material was identified by Wanderlei do Amaral and exsiccates were prepared and deposited in the Herbarium of the Faculdades Integradas Espírita (HFIE), where they were herborized (HFIE ‐ 8.820) [34].

### 4.2 Zebrafish

Zebrafish (*Danio rerio*) (aged 90 to 120 days; 0.4 ± 0.1 g, 3.5 ± 0.5 cm), wild, of both sexes, were purchased from a local store (Fortaleza, CE). The fish were kept in a glass aquarium (30 × 15 × 20 cm) with a 10 L capacity (*n* = 3 / L), using chlorinated water (ProtecPlus) and an air pump with submerged filters, at a temperature of 25°C and pH 7.0, under a 14‐10 h light/dark circadian cycle. The fish were fed with Spirulina ad libitum 24 h before the experiments. Prior to drug administration, the animals were anesthetized in cold water and after the experiments, they were euthanized by immersion in cold water (2 and 4°C) for 1 min until they ceased opercular movement. The study was approved by the Ethics Committee for Animal Use of the State University of Ceará (CEUA‐UECE; no. 04983945/2021) and complied with the Ethical Principles of Animal Experimentation. Adult zebrafish (males and females) were randomly assigned to experimental groups (*n* = 6/group), with both sexes represented in all treatments. Behavioral assessments were performed under blind conditions [23]. Each experimental protocol was conducted once, using six individual fish per treatment group. The animals originated from the same holding tank and were randomly allocated to the experimental groups. All animals that completed the experimental protocols were included in the statistical analysis, with no exclusions made after allocation. The sample size was based on zebrafish behavioral protocols previously established by the research group and was determined to ensure experimental consistency while minimizing animal use. Sex was not included as an independent factor in the statistical analysis as the study was designed to evaluate the overall pharmacological response rather than sex‐specific effects. Following selection, the fish were anesthetized in cold water, transferred to a damp sponge, and treated with 20 µL of the sample via intraperitoneal injection at varying doses (4, 20, and 40 mg/kg), or with Diazepam (4 mg/kg), 3% DMSO (vehicle control), or fluoxetine.

Throughout the experimental procedures, the animals were continuously monitored for behavioral and physical signs of distress. Handling and the duration of the experiment were minimized, and standardized environmental conditions were maintained to reduce unnecessary stress. At the end of the predefined experimental assessments, the animals were euthanized via rapid cooling by immersion in an ice–water bath, in accordance with the institutionally approved protocol [15]

### 4.3 Acute Toxicity 96 h

The fish (*n* = 6 / group) were treated following the general protocol instructions and allowed to rest for mortality analysis over a period of 96 h, recording the number of dead fish in each group every 24 h [27]. The lethal dose capable of killing 50% of the animals (LD_50_) was determined using the Trimmed Spearman–Karber mathematical method with a 95% confidence interval.

### 4.4 Assessment of Locomotor Activity (Open Field Test—OFT)

The open field test was conducted to assess the presence or absence of changes in motor coordination in animals [20], whether due to anxiolytic effects and/or muscle relaxation. The animals (*n* = 6/group) were treated as described in the Zebrafish section. Diazepam (Dzp; 4 mg/kg) and 3% DMSO (vehicle control) were used as controls. After 30 min of treatment, the animals were individually placed in glass Petri dishes (10×15 cm; with quadrants on the bottom), containing the same water as the aquarium. The number of line crossings was recorded over a 0–5 min period.

### 4.5 Anxiolytic Assessment (Light/dark Test—LDT)

The anxiety behavior of an animal can be observed through the light/dark test. Similar to rodents, zebrafish naturally avoid brightly lit areas [14, 35]. The experiment was conducted in a glass aquarium (30 cm x 15 cm x 20 cm) divided into a light area and a dark area. The aquarium was filled with tap water up to 3 cm, simulating a shallow new environment different from the conventional aquarium, capable of inducing anxiety behaviors. The sample was administered to the animals (*n* = 6/group) as mentioned in the Zebrafish section. The negative and positive control groups consisted of 3% DMSO (vehicle control) and a Diazepam solution (4 mg/kg), respectively. After 30 min, the animals were individually placed in the light zone, and the anxiolytic effect was measured based on the time spent in the light zone of the aquarium during a 5‐min observation period [20].

### 4.6 Assessment of GABAergic Neuromodulation

GABAergic neuromodulation involved in the anxiolytic effect was performed through pre‐treatment with flumazenil (GABAA antagonist) before the light/dark test [29]. The fish (*n* = 6/group) were pre‐treated with flumazenil (4 mg/kg; 20 µL; i.p.). After 15 min, the dose with the highest anxiolytic efficacy found in the previous test was administered. 3% DMSO (vehicle control; 20 µL; i.p.) was used as a negative control. After 30 min of treatment, the animals were subjected to the light/dark test.

Siebel et al. [30, 31] investigated the reversal of pentylenetetrazole (PTZ)‐induced seizures. Adult zebrafish (*n* = 6/group) received intraperitoneal (i.p.) injections of oils (4, 20, and 40 mg/kg; 20 µL), Diazepam (4 mg/kg; 20 µL; i.p.), or vehicle control (3% DMSO; 20 µL; i.p.). After 30 min, animals were exposed to 7.5 mM PTZ in a 250 mL beaker, and convulsive behavior was assessed in three stages: increased swimming activity (I), whirlpool behavior (II), and clonus‐like seizures followed by postural loss (III). At the end of the experimental procedures, the animals were euthanized by rapid chilling through immersion in an ice‐water bath (≤4°C), according to the institutionally approved protocol and established recommendations for adult zebrafish euthanasia [24].

### 4.7 Pentylenetetrazole (PTZ)‐Induced Seizures

Anticonvulsant activity was evaluated using the PTZ‐induced seizure model. Adult zebrafish (*n* = 6/group) were treated with OEPL37 (4, 20, or 40 mg/kg; 20 µL; i.p.), diazepam (4 mg/kg; 20 µL; i.p.), or vehicle (3% DMSO; 20 µL; i.p.). After 30 min, the animals were individually exposed by immersion to PTZ (10 mM), prepared by dissolving 0.3467 g in 250 mL of distilled water at room temperature. Latencies to three seizure stages were recorded: stage I, increased swimming activity; stage II, whirlpool‐like swimming behavior; and stage III—clonus‐like seizures followed by loss of posture [8]. At the end of the experimental procedures, the animals were euthanized by rapid chilling through immersion in an ice‐water bath (≤4°C).

### 4.8 Molecular Docking Simulations

The two‐dimensional structure of compounds DFX, DHF, ERM, and SEL was plotted and rendered using the academic license program MarvinSketch, version 25.5.0, CERTARA (https://www.certara.com/marvin/) and saved in .mol format for three‐dimensional optimization. The ligands in their three‐dimensional coordinates were optimized using the Merck Molecular Force Field (MMFF94) method [36], in the Avogadro software, version 1.2.0 (https://avogadro.cc/), preparing the ligands for molecular docking simulations.

The virtual repository RCSB Protein Data Bank (PDB) (https://www.rcsb.org/) was used to obtain the structure of the GABAA receptor, identified by “CryoEM structure of human full‐length alpha1beta3gamma2L GABA(A)R in complex with diazepam (Valium), GABA and megabody Mb38,” classified as a membrane receptor in the *Homo sapiens* and *Escherichia coli* organisms at resolution of 3.58 Å (PDB Code 6HUP). Subsequently, the structure was submitted to the program AutoDock Tools version 1.5.6—MGL Tools (https://autodock.scripps.edu/) to be prepared and used in molecular docking simulations.

The protein preparation included the removal of water molecules (H_2_O), the addition of polar hydrogens (in O and N atoms) to the side chains of the residues according to the protonation state at physiological pH (7.4), the Gasteiger and Kollman charges were calculated, and the grid‐box that defined the three‐dimensional space of the molecular docking simulations was plotted. The grid‐box was adjusted to encompass the entire conformational space of the protein, set at dimensions *x* = 92 Å, *y* = 94 Å, and *z* = 131 Å on the axes *x* = 134.4, *y* = 135.7, and *z* = 133.7.

Finally, the AutoDock Vina version 1.2.0 code (https://vina.scripps.edu/) was configured to run a cycle of 50 independent simulations of 20 poses, of the multi‐ligand virtual screening model [37], using the Lamarckian Genetic Algorithm (LGA), with a degree of exhaustiveness = 64 [38].

The validation protocol for the molecular docking simulations was performed by redocking DZP, an agonist co‐crystallized to the PDB Code 6HUP model. The best‐pose selection criteria include alignment between Root Mean Square Deviation (RMSD) less than 2.0 Å and an affinity energy score less than ‐6.0 kcal/mol [39].

### 4.9 PK/PD Study and Toxicity Prediction

The quantitative estimate of drug‐likeness (QED) protocol was applied to evaluate the contribution of the physicochemical properties of the pharmacokinetic and pharmacodynamic (PK/PD) parameters of the compounds DFX, DHF, ERM, and SEL, as shown in Equation 1:

(1) $$\mathrm{QED}=\exp\;\left(\frac{\sum_{i=1}^{n}w_{i}\ln\;(d_{i})}{\sum_{i=1}^{n}w_{i}}\right)$$

where *w* is the weighting factor that each physicochemical property *i* has within the desirability functions (d), defined by: molecular weight (MW) 200–600 g/mol, lipophilicity (logP) ≤ 3.0, H‐bond acceptors (nHA) 2–10, H‐bond donors (nHD) 0–5, topological polar surface area (TPSA) 20‐130 Å2, number of rotatable bonds (nRot) 0–10, number of rings (nRing) 0–3, and structural alerts = 0, and the sum results in a score that varies from 0.0 (poor drug‐likeness) to 1.0 (optimal drug‐likeness) [40].

The chemical structures of the major derivatives DFX, DHF, ERM, and SEL were plotted for surface analysis of molecular lipophilicity potential (MLP) using the academic license program MarvinSketch, version 25.5.0, CERTARA (https://www.certara.com/marvin/), and the physicochemical properties were calculated using the predictive tool ADMETlab 3.0 (https://admetlab3.scbdd.com/). Then, PK/PD properties were predicted using an ensemble approach that combines multiple platforms: ADMETlab 3.0 (https://admetlab3.scbdd.com/), ADMET‐AI (https://admet.ai.greenstonebio.com/), pkCSM (https://biosig.lab.uq.edu.au/pkcsm/), ADMETboost (https://ai‐druglab.smu.edu/admet), and SwissPK/PD (http://www.swissadme.ch/). The prediction of toxicity properties was made from the online server ADMETboost (https://ai‐druglab.smu.edu/admet), where toxicity endpoints related to drug‐induced liver injury (DILI), Ames mutagenicity, cardiotoxicity, and cytochrome P450 (CYP450)‐dependent metabolism were predicted using an artificial intelligence module to estimate organic toxicity properties that were related to the prediction of acute oral toxicity by estimating the lethal dose (LD_50_) in rats using the STopTox tool (https://stoptox.mml.unc.edu/).

### 4.10 ADMET Data

The data from the ADME Properties and Toxicity prediction generated internally using the servers were: (a) apparent passive permeability (P_app,A→B_), using cell models that express human P‐glycoprotein (P‐gp), namely Caco‐2 and MDCK; (b) human oral bioavailability (%F) and human intestinal absorption (HIA); (c) plasma and CNS distribution, via the BBB permeation coefficient (logBB) and plasma protein binding (PPB); and (d) human liver microsome (HLM) stability and metabolic stability, expressed as unbound intrinsic clearance (CL_int,u_). The reported organic toxicity data are: (a) probability of blocking human Ether‐a‐go‐go‐Related Gene (hERG) channels; (b) Ames test mutagenicity; (c) Drug‐Induced Liver Injury (DILI); and (d) lethal dose in rats (LD_50_).

### 4.11 Statistical Analysis

The results were expressed as mean ± standard error of the mean (SEM) for each group of six animals. Data normality was assessed using the Shapiro–Wilk test, and homogeneity of variances was evaluated using Levene's test. Differences among groups were analyzed by one‐way analysis of variance (ANOVA), followed by Tukey's multiple‐comparisons test. For the GABAergic neuromodulation experiment, two‐way ANOVA followed by Tukey's multiple‐comparisons test was used. Statistical significance was considered at *p* < 0.05.

## 5 Conclusion

*Nectandra grandiflora* essential oil showed an anxiolytic‐like effect at 4 mg/kg in adult zebrafish, and the attenuation of this response by flumazenil supports the involvement of GABA receptor‐mediated neuromodulation. In contrast, the essential oil did not demonstrate anticonvulsant efficacy against PTZ‐induced seizures at the evaluated doses. Molecular docking identified SEL as the compound with the most favorable predicted binding affinity to the GABA_A_ receptor, while ADMET analysis suggested potentially favorable pharmacokinetic properties; however, both findings require experimental validation to confirm their biological and pharmacological relevance. No evident acute toxicity was observed under the experimental conditions and doses evaluated. Overall, the findings support the neuropharmacological potential of *N. grandiflora* essential oil and provide a basis for further investigation of its bioactive constituents and mechanisms of action.

## Author Contributions

**Letícia Kelly Mesquita Rodriguesa**: investigation; formal analysis; writing – original draft. **Hélcio Silva dos Santos**: co‐supervision; writing – review and editing. **Luiz Everson da Silva**: project administration; conceptualization. **Wanderlei do Amaral**: funding; formal analysis; writing – review and editing. **Jenifer Priscila de Araujo**: methodology; formal analysis. software; formal analysis; writing – review and editing. **Jane Eire Silva Alencar de Menezes**: methodology; formal analysis. **Dayane Crystina da Silva Ceneviva**: software, visualization. **Jessica Bezerra Maciel**: methodology; formal analysis. **Márcia Machado Marinho**: methodology, writing – review and editing. **Emmanuel Silva Marinho** and **Matheus Nunes da Rocha**: supervision.

## Funding

Helcio Silva dos Santos acknowledges financial support from CNPq (Grant 304807/2026‐5) and FUNCAP‐INTERNACIONALIZAÇÃO (Grant ITR‐0214‐00060.01.00/23). Emmanuel Silva Marinho acknowledges financial support from FUNCAP (Grant#: FPD‐0213‐00369.01.00/23) and CNPq (Grant#: 309349/2025‐7).

## Conflicts of Interest

The authors declare no conflicts of interest.

## Data Availability

The data that support the findings of this study are available from the corresponding author upon reasonable request.
